# Periodontitis: A Plausible Modifiable Risk Factor for Neurodegenerative Diseases? A Comprehensive Review

**DOI:** 10.3390/ijms25084504

**Published:** 2024-04-19

**Authors:** Adelina S. Plachokova, Jolijn Gjaltema, Eliza R. C. Hagens, Zahra Hashemi, Tim B. A. Knüppe, Thomas J. M. Kootstra, Anita Visser, Bastiaan R. Bloem

**Affiliations:** 1 Department of Dentistry, Radboud University Medical Center, 6525 EX Nijmegen, The Netherlands; jolijn.gjaltema@radboudumc.nl (J.G.); eliza.hagens@radboudumc.nl (E.R.C.H.); zahra.hashemi@radboudumc.nl (Z.H.); tim.knuppe@radboudumc.nl (T.B.A.K.); tom.kootstra@radboudumc.nl (T.J.M.K.);; 2 Department of Gerodontology, Center for Dentistry and Oral Hygiene, University Medical Center Groningen, University of Groningen, 9700 RB Groningen, The Netherlands; 3 Radboud University Medical Centre, Donders Institute for Brain, Cognition and Behavior, Department of Neurology, Centre of Expertise for Parkinson and Movement Disorders, 6525 GA Nijmegen, The Netherlands; bas.bloem@radboudumc.nl

**Keywords:** periodontitis, neurodegenerative diseases, Alzheimer’s disease, Parkinson’s disease, neuroinflammation, risk factor

## Abstract

The aim of this comprehensive review is to summarize recent literature on associations between periodontitis and neurodegenerative diseases, explore the bidirectionality and provide insights into the plausible pathogenesis. For this purpose, systematic reviews and meta-analyses from PubMed, Medline and EMBASE were considered. Out of 33 retrieved papers, 6 articles complying with the inclusion criteria were selected and discussed. Additional relevant papers for bidirectionality and pathogenesis were included. Results show an association between periodontitis and Alzheimer’s disease, with odds ratios of 3 to 5. A bidirectional relationship is suspected. For Parkinson’s disease (PD), current evidence for an association appears to be weak, although poor oral health and PD seem to be correlated. A huge knowledge gap was identified. The plausible mechanistic link for the association between periodontitis and neurodegenerative diseases is the interplay between periodontal inflammation and neuroinflammation. Three pathways are hypothesized in the literature, i.e., humoral, neuronal and cellular, with a clear role of periodontal pathogens, such as *Porphyromonas gingivalis*. Age, gender, race, smoking, alcohol intake, nutrition, physical activity, socioeconomic status, stress, medical comorbidities and genetics were identified as common risk factors for periodontitis and neurodegenerative diseases. Future research with main emphasis on the collaboration between neurologists and dentists is encouraged.

## 1. Introduction

### 1.1. Clinical Relevance

The conception of this narrative review started in the dental office and was inspired by real patients suffering from both neurodegenerative diseases, such as Parkinson’s disease (PD), and periodontitis. Unfortunately, to date, neurodegenerative diseases cannot be cured, whereas periodontitis is treatable. If both diseases are etiologically and pathogenically linked, knowledge on periodontal disease and its treatment might bring us close to a solution on how to slow down or prevent further progression of neurodegenerative diseases. In view of this “clinically born” hypothesis, three research questions appear highly relevant to be studied in the scientific literature:

- Which neurodegenerative diseases are reported to be associated with periodontitis, and what is the evidence for this association?
- Are patients with periodontitis at greater risk of developing neurodegenerative diseases and vice versa? Which common risk factors do periodontitis and neurodegenerative diseases share?
- What is the plausible mechanistic link for this association?

### 1.2. Scientific Background

In recent decades, the prevalence of neurodegenerative diseases such as Alzheimer’s disease (AD) and Parkinson’s disease (PD) has been rapidly increasing, with PD being the world’s fastest growing neurological disorder [1,2]. This is of major health and public concern, especially due to the lack of cure up to this moment. Therefore, identifying possible risk factors that might help to prevent or to slow down the development and/or progression of neurodegenerative diseases is of utmost importance. Of particular interest are risk factors that can be affected, i.e., are modifiable. Neuroinflammation triggered by systemic inflammation is suspected to play a role in the pathogenesis of non-hereditary neurodegenerative diseases. Periodontitis is a very common local inflammatory disease that can induce systemic inflammation. In view of that, it appears relevant to explore and discuss whether periodontitis has the potential to qualify as a risk factor for neurodegenerative diseases [3].

### 1.3. Periodontitis

**Figure 1:**
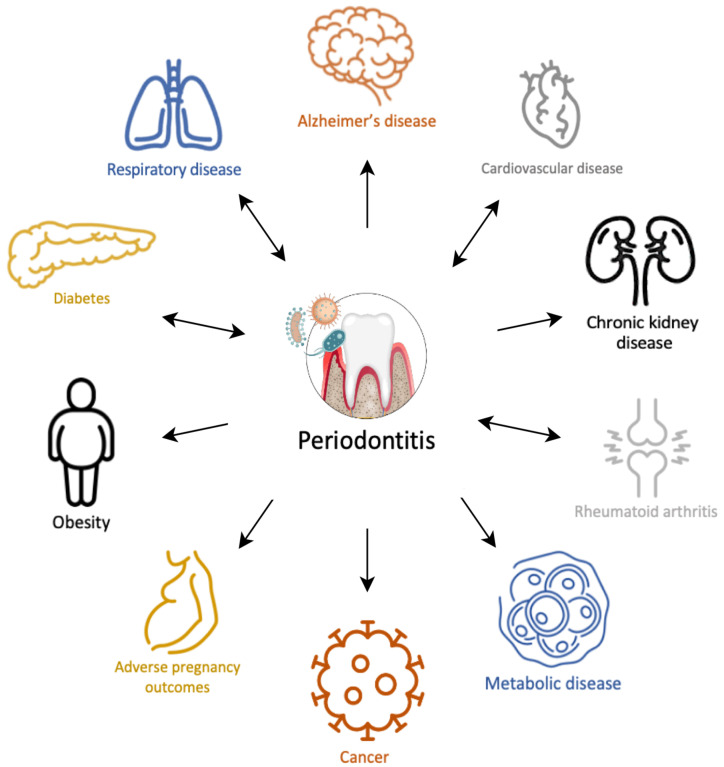
Periodontitis and some of its comorbidities [6].

Periodontitis is a multifactorial, microbiome-driven inflammatory disease of the tooth-attachment apparatus. It affects between 45 and 50% of the population worldwide, and its incidence increases with age [4]. Clinically, periodontitis is characterized by dental plaque and calculus, gingival bleeding, periodontal pockets, tooth attachment loss and bone loss, which ultimately leads to tooth loss. In periodontitis, microbial dysbiosis and chronic inflammation co-develop in a reciprocally reinforced manner [5]. Briefly, periodontal pathogens (i.e., communities of mainly Gram negative anaerobes) induce gingival inflammation, which in turns creates local conditions (i.e., periodontal pockets) that are suitable for the survival and expansion of these pathogens, which on their turn reinforce and enhance the gingival inflammation. Hence, a vicious circle of low-grade chronic inflammation develops. This low-grade local inflammation is known to trigger systemic inflammation via direct mechanisms (i.e., bacteria, their toxic products and local cytokines, such as IL-6, IL-1β and TNF-α enter the bloodstream) or indirect mechanisms (via immune host response; innate and acquired immunity). The consequences could be detrimental and may occur at distant sites, as suggested by the multiple comorbidities of periodontitis (Figure 1). Figure 1 presents an overview of the systemic diseases to which periodontitis has been associated, and is based on data already published [6].

Currently, there is some evidence to support the independent associations between severe periodontitis (i.e., global prevalence 19% according to the World Health Organization) and non-communicable diseases—in particular cardio-vascular disease (CVD) and diabetes mellitus (DM) [7]. Several consensus reports have been published and raised awareness among the medical community that periodontitis is a treatable and preventable disease, hence a potentially modifiable risk factor of systemic diseases [4,8,9]. Conventional periodontal treatment is straightforward, and it involves 4 steps. Step 1 is guiding behavior changes by motivating the patient and risk factor control. Step 2 is considered cause-related therapy, aimed at controlling (reducing/eliminating) the subgingival biofilm and calculus (subgingival instrumentation). Step 3 is focused on treating areas that do not respond adequately to the second step of therapy. Step 4 is supportive periodontal care, aimed at maintaining periodontal stability in all treated periodontitis patients. These four steps are based on scientific evidence for efficacy and are part of international recommendations also used for patients with periodontitis and comorbidities [10].

According to the latest microbiological hypotheses in the etiology of periodontitis, *Porphyromonas gingivalis* (*P. gingivalis*) is the “keystone-pathogen” that drives the process of oral dysbiosis [11,12,13]. Although these hypotheses are still theoretical concepts, *P. gingivalis* is the most studied periodontal pathogen. Previously, we explored the role of *P. gingivalis* in view of the association between periodontitis and CVD [14]. We found out that *P. gingivalis* can induce long-term activation of human monocytes in vitro and has the potential to be involved in the process of trained immunity in patients with periodontitis. Our findings may also be relevant in regard to the association between periodontitis and neurodegenerative diseases. Emerging studies suggest that trained immunity might also play a role in the pathogenesis of AD and PD [15,16]. It may contribute to an increase in uncontrolled inflammation and sustain the neuroinflammatory cycle as described [17].Trained immunity is a recent revolutionary concept in immunology that has shown that cells of the innate immunity (i.e., monocytes and macrophages) can also build up memory after encountering a pathogen [18]. As a result, they acquire a hyper-responsive phenotype via epigenetic changes and can induce increased production of cytokines, which is the hallmark of inflammation. Trained immunity offers protection against recurrent infections. However, in diseases, in which chronic inflammation contributes to disease pathophysiology, such as periodontitis and probably AD and PD, it could be detrimental. It has to be emphasized that *P. gingivalis*, notorious for its gingipains, toxic proteases, and lipopolysaccharides (LPS), has already been investigated extensively in the etiopathogenesis of AD [19,20]. Indeed, a PubMed search on *P. gingivalis* and AD retrieved 116 relevant articles, whereas the same search for *P. gingivalis* and PD showed only 10 papers.

### 1.4. Periodontitis as Plausible Modifiable Risk Factor for Systemic Diseases

**Figure 2:**
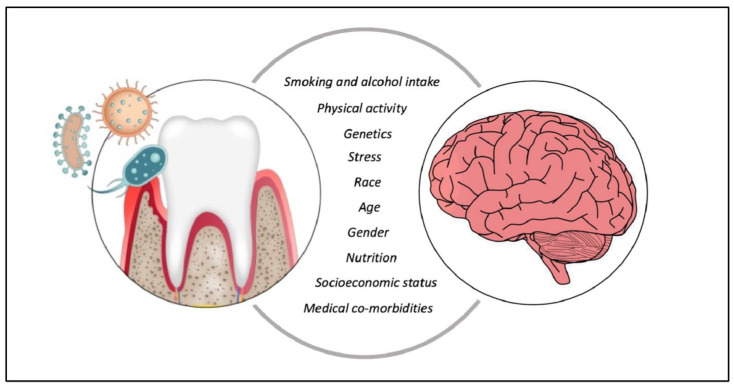
Common risk factors between neurodegenerative diseases and periodontitis. This schematic presentation is based on data reported in a systematic review with a meta-analysis [34].

As mentioned previously, periodontitis is epidemiologically associated with other inflammation-driven systemic diseases and conditions, including cardio-metabolic, autoimmune diseases, respiratory infections, and certain cancers (Figure 2) [6]. These associations may partially be causal (e.g., periodontitis could initiate or aggravate comorbid conditions) as suggested by animal studies and some interventional trials, which show that treatment of periodontitis reduces systemic inflammation and surrogate markers of comorbid diseases [6,21,22]. For instance, patients with type 2 DM seem to benefit from professional mechanical debridement in combination with sustained effective oral health care. Levels of HbA1C reduction obtained within the short term after periodontal treatment are comparable to those achieved by adding a second drug into the patients pharmacological management [8]. Recently, a systematic review evaluated the methodological quality of 52 reviews on the effect of non-surgical periodontal treatment (i.e., step 2) on systemic disease outcomes [21]. It has to be noted that in this study, no review that focused on neurodegenerative diseases and periodontitis was included.

In addition, there is some evidence for bidirectional relationships between periodontitis and its comorbidities, e.g., periodontitis and DM [9,23]. The concept of “mechanistic causality” between periodontitis and its comorbidities appears to be biologically plausible and clinically consistent [24]. However, this concept has not be extensively explored for periodontitis and neurodegenerative diseases.

In view of all of the above mentioned, it appears reasonable and relevant to explore the association between periodontitis and neurodegenerative diseases, whether it is uni- or bidirectional and reveal the plausibility for “mechanistic causality”. Therefore, the aim of this comprehensive literature review is to summarize the recent literature on this topic, discuss periodontitis as a potential modifiable risk factor and to provide insights into the plausible pathogenesis.

## 2. Which Neurodegenerative Diseases Are Reported to Be Associated with Periodontitis, and What Is the Evidence for This Association?

To answer this question PubMed, Medline and EMBASE were searched for systematic reviews and meta-analyses published during the last 5 years. Articles on periodontitis (also called periodontal disease) in association with neurodegenerative diseases such as AD, PD, amyotrophic lateral sclerosis, frontotemporal dementia and Huntington’s disease were included. Exclusion criteria were: (1) articles in languages other than English, German or Dutch, (2) animal studies, (3) not listing the etiology of dementia (e.g., no clear distinction between vascular and neurodegenerative causes of dementia) and (4) no availability of full text. Other rare neurodegenerative diseases not handled in this review include multiple system atrophy, progressive supranuclear palsy, motor neuron disease, spinocerebellar ataxia and Wilson’s disease. The search strategy is presented below.
Periodont*[title] AND (neurodegen*[title/abstract] OR Parkinson*[title/abstract] OR Alzheimer*[title/abstract] OR (ALS[title/abstract] OR amyotrophy*[title/abstract]) OR “frontotemporal dementia”[title/abstract] OR Huntington*[title/abstract]) AND (review[title] OR “meta-analysis”[title])33Periodont*[title] AND (neurodegen*[title/abstract] OR Parkinson*[title/abstract] OR Alzheimer*[title/abstract] OR (ALS[title/abstract] OR amyotrophy*[title/abstract]) OR “frontotemporal dementia”[title/abstract] OR Huntington*[title/abstract]) AND (review[title] OR “meta-analysis”[title]) **Filters:** in the last 5 years25

In total, 33 systematic reviews and meta-analyses on the association between periodontitis and neurodegenerative diseases were retrieved. After screening on title and abstract, and applying the inclusion and exclusion criteria, six articles remained for further analysis and discussion. The selected studies with summary of their main findings are presented in Table 1.

In our research strategy, we applied search terms for different types of neurodegenerative diseases in order to identify as many papers as possible on this subject. However, systematic reviews were detected mainly on AD, i.e., 5 out of 6, followed by PD, Multiple Sclerosis (MS) and mild cognitive impairment (MCI). A possible explanation could be that AD is the most common neurodegenerative disease worldwide, and hence the most extensively studied one. In addition, periodontitis and AD might share relatively more similarities in their pathogenesis compared to periodontitis and other neurodegenerative conditions. All studies reported a positive association with periodontitis, including MS and MCI (i.e., OR between 1.60 and 2.32 for MCI [27]). Further in this review, we will focus only on the mostly studied neurodegenerative diseases, i.e., AD and PD.

### 2.1. Alzheimer’s Disease (AD)

All studies found a significant positive association between periodontitis and AD. Reported odds ratios for severe periodontitis and AD varied from OR = 2.98 (95% CI 1.58–5.62) in Leira et al. to OR = 4.89, 95% CI 1.60–14.97) in Hu et al. [25,27]. In addition, the presence of periodontitis at baseline was associated with a six-fold increase in rate of cognitive decline over a 6-month follow-up (ADAS-Cog mean change = 2.9 ± 6.6) in Borsa et al. [19]. The latter is a very interesting and highly clinically relevant finding that should be further investigated. It is also a call for action for more interventional studies on periodontitis in patients with AD to explore the potential of periodontal treatment to prevent or to slow down the progression of AD.

In the presented systematic reviews, specific periodontal bacteria were studied for a possible link with AD. Hu et al. found an increase in *Fusobacterium nucleatum (F. nucleatum)* in AD patients (adjusted *p* = 0.02), and its incidence was linked to *Campylobacter rectus (C. rectus)* and *P. gingivalis* (adjusted HR = 1.22 (1.04–1.43), *p* = 0.012) in addition to *Actinomyces naeslundii (A. naeslundii)* (crude HR = 2.0 (1.1–3.8) [27]. Post mortem studies included in the review of Mao et al. demonstrated that the classical periodontal pathogens, i.e., *P. gingivalis*, *Tanerella forsythia (T. forsythia)* and *Treponema denticola (T. denticola)* known as the Socransky’s “red complex” were found significantly more often in brain tissue of patients with AD [28,29]. The “red complex” bacteria are associated with severe form of periodontitis, and usually found together in periodontal pockets [30]. Although this is an intriguing finding and supports the pathogenic link between periodontitis and AD, it should be interpreted with caution. Further research in preclinical models is necessary to give extra insight/confirm whether these pathogens are “promoters” and not only “passengers”. Current indications from animals studies are that *P. gingivalis* and *F. nucleatum* could be “promoters” [20,31]. These findings together with clinical results showing that mechanical debridement in periodontitis could reduce the bacterial load [22] is a call for more interventional trials in AD patients.

### 2.2. Parkinson’s Disease (PD)

Out of six retrieved studies, only one systematic review with meta-analysis was entirely focused on the association between PD and periodontitis. This review did not find a significant association. However, it did find a correlation between the risk of developing PD and poor oral health [29]. PD patients presented a higher prevalence of periodontitis and other periodontal diseases, as well as poor oral hygiene compared to healthy individuals. In the meta-analysis, PD patients exhibited higher levels of periodontal pocket depth (SMD = 1.10, 95% CI 0.53–1.67), clinical attachment level (SMD = 1.40, 95% CI 0.55–2.26), plaque index (SMD = 0.81, 95% CI 0.22–1.39), and Oral Health Impact Profile-14 score (SMD = 0.91, 95% CI 0.33–1.49) compared to healthy controls. It has to be noted that the authors themselves admitted that the weak association between PD and the risk of periodontitis they had found may be unreliable and should be interpreted with caution. The reason was that their conclusion was based on the results of only one study, i.e., Mendelian randomization (MR) study, as no other studies examined the association between PD and periodontitis. Obviously, there is a huge knowledge gap in this area that should be addressed.

### 2.3. Microbiological Factors and Neurodegenerative Diseases

In addition to the presented studies, the outcomes of an umbrella review with quality-grade evidence of systematic reviews and meta-analysis on this topic require special attention. Wang et al. comprehensively summarized and evaluated the associations between microbiological factors and the risk of neurodegenerative disorders [32]. Out of 37 unique associations they had studied, only 4 associations had above the medium level of evidence. Among them, *Helicobacter pylori* (*H. pylori*), infection, and bacteria were found to be associated with PD. It addition, periodontitis was identified a risk factor for all types of dementia. Based on the results of this umbrella review, eradication of *H. pylori* and aggressive treatment of periodontitis has been advocated by the authors as a possible way to prevent PD and dementia, respectively. We completely agree and support their statement for action. The authors further concluded that despite the immense amount of evidence linking microbial factors to neurodegenerative diseases, these associations appear not necessarily causal, and the evidence level was low due to heterogeneity of the studies. Therefore, more effective studies were encouraged.

### 2.4. Methodological Issues in Original Studies on Association

To better understand the above presented research results on associations between periodontitis and neurodegenerative diseases, and to become aware of some methodological issues frequently encountered, two other systematic reviews with meta-analyses are important to consider. Larvin et al. explored the study factors that affect the association between cognitive disorders and periodontitis [33]. Based on a meta-analysis of 39 studies (e.g., 13 cross-sectional and 26 longitudinal), the authors concluded that the prevalence and risk estimates of cognitive disorders in association with periodontitis can be influenced by gender, the disease classification of periodontitis and its severity. Despite these issues, having periodontitis was associated with increased risks of cognitive disorders (cognitive decline-RR = 1.33, 95% CI = 1.13–1.55; dementia/AD-RR = 1.22, 95% CI = 1.14–1.31) and the risk of cognitive decline increased with the severity of periodontitis (moderate-[RR] = 1.14, 95% confidence interval [CI] = 1.07–1.22; severe-RR = 1.25, 95% CI = 1.18–1.32). Further research taking these study factors into consideration to draw robust conclusions was advocated.

Dziedzic paid special attention to the confounding factors in studies on the association between neurodegenerative diseases and periodontitis [34]. The main research question was whether periodontitis was a risk factor for age-related cognitive impairment. The primary and residual confounders from 17 clinical studies with sample sizes ranging from 85 to 262, 349 subjects and follow-up between 2 and 32 years were explored and evaluated. A remarkable variation related to the treatment of confounders was observed in addition to the substantial differences in the study designs and research methods. Nevertheless, their meta-analysis showed that the presence of periodontitis was associated with an increased incidence of cognitive impairment (OR = 1.36, 95% CI 1.03–1.79), particularly dementia (OR = 1.39, 95% CI 1.02–1.88) and AD (OR = 1.03 95% CI 0.98–1.07)). However, it was noted that a considerable heterogeneity of synthesized data (I^2^ = 96%) and potential publication bias might have affected the results. The author concluded that while there is a moderate statistical association between periodontitis and dementia, as well as AD, the risk of bias in the evidence prevents conclusions to be drawn on the role of periodontitis as a risk factor for age-related cognitive impairment.

## 3. Are Patients with Periodontitis at Greater Risk of Developing Neurodegenerative Diseases and Vice Versa? Which Common Risk Factors Do Periodontitis and Neurodegenerative Diseases Share?

To answer these research questions comprehensively, additional relevant papers on AD and PD were considered in addition to the included systematic reviews and meta-analyses.

### 3.1. Bidirectional Link and Reverse Causality

Patients with periodontitis seem to present a higher risk for developing AD (i.e., 3 to 5 times), and the risk of AD incidence doubles within 10 years from the time of periodontal disease diagnosis [25,27,35]. In view of PD, the risk is not completely clear. This has to do with the lack of research [29] and the contradictory results from current epidemiological studies. The majority of the epidemiological studies come from Asian countries, Taiwan and Korea, which have similar healthcare systems. While cohort Taiwanese studies indicate elevated risk for PD, i.e., adjusted HR = 1.431, 95% CI [1.141–1.794], *p* = 0.002) and Parkinsonism in patients with periodontitis [36,37], Korean studies report either no [38,39] or weak associations [40]. Up to 2 years, no increased likelihood to develop PD was observed [39]; up to 8 years, severity of periodontitis and incidence of PD were significantly associated [40]. Patients with mild periodontitis had a 7.6% to 9.5% higher risk for PD, whereas the highest risk was reported for periodontitis combined with metabolic syndrome (i.e., 16.7% higher risk).

Patients with AD and PD may have poor oral health as a consequence of their cognitive decline and psychical disability, hence they may be more likely to develop periodontitis (i.e., reverse causality) [29,41]. Maintaining good oral hygiene, which includes daily toothbrushing, interdental cleaning, and avoiding excessive sweet or sour foods, can be challenging for both patient groups. Moreover, a decline in dental care could elevate the risk of periodontal disorders [42]. Verhoeff et al. found that poor oral health of PD patients was associated with a longer disease duration, higher disease severity, and more prescribed medications [43]. Pruntel et al. in a systematic review explained the associations between oral health diseases and AD with among others the role of pathogens and role of inflammatory mediators [44]. In a large-scale study in cohorts of patients with dementia and AD with no periodontitis at baseline, the risk of developing periodontitis up to 10 years was calculated [45]. The risk was found to be age dependent, and independent of systemic confounding factors. The AD patients had relative risk (1.531, 95% CI = 1.209 to 1.939) and adjusted HR (1.667, 95% CI = 1.244 to 2.232; log-rank test *p* = 0.0004) that was significantly higher than the non-AD cohort.

Despite the presented suggestions for a bidirectional relationship for AD and speculations about PD, it is still unclear whether periodontitis or AD/PD develop first. Moreover, it seems very difficult or even impossible to prove causality due to the complex nature of both periodontitis and neurodegenerative diseases, and the common risk factors.

When we go back to the literature on the potential bidirectional link between periodontitis and neurodegenerative diseases, we cannot ignore the contradicting statements and conclusions from different systematic reviews and meta-analysis. Chen et al. stated that the bidirectional relationship between PD and periodontitis had not undergone sufficient systematic assessment to draw definitive conclusions [36]. Supporting this conclusion, Dziedzic pointed out that there was not enough strong clinical evidence to support causality [34]. In contrast, in another systematic review with meta-analysis, Nadim et al. concluded that a significant association between periodontitis and the risk of dementia exists [46]. The authors even suggested that globally reducing periodontitis could possibly reduce the increasing incidence of dementia. Said-Sadir et al. supported the conclusion drawn by Nadim et al. and advocated for further research on the responsible association, as this is not clear yet [47].

### 3.2. Periodontitis as Plausible Modifiable Risk Factor for AD and PD

Studies on periodontitis and periodontal therapy as a modifiable risk factor for neurodegenerative diseases is an emerging research area. Since periodontal treatment reduces inflammation in oral tissues, it has been hypothesized that it may positively affect systemic outcomes by reducing inflammation in the body [21]. We were able to identify a few studies that support the plausibility of the concept of periodontal treatment modifying the disease outcomes in neurodegenerative diseases, and present them below.

Schwahn et al. investigated the relationship between periodontal treatment and preclinical AD [48]. In a trial-emulation approach, they found that periodontal treatment had a favorable effect on AD-related brain atrophy (−0.41; 95% confidence interval: −0.70 to −0.12; *p* = 0.0051). In addition, there is some epidemiological evidence suggesting that eradication of *P. gingivalis*, among others, could be an effective way to delay the onset of AD, calling for randomized clinical trials [49]. This suggestion was based on data with 26 years of follow-up showing evidence for an association between periodontal pathogens and AD, which was stronger for older adults, i.e., individuals above 55 (HR = 1.06) or 65 years (HR = 1.12). Relevant and promising findings from systematic reviews on AD are that 32% of the MCI cases would convert into AD within 5 years [50] and this process seems reversible, overall rate 10% [51]. Therefore, controlling the development of MCI is considered important for AD prevention. Since there is some evidence for an association between MCI and periodontitis, i.e., OR = 2.32 [27], and periodontitis is treatable, it appears plausible to try to prevent cognitive impairment, hence AD, by early intervention to treat periodontitis.

In view of PD, the first nationwide, population-based case–control study of Taiwan showed that individuals without periodontitis, who underwent dental scaling for 5 consecutive years had a significantly decreased risk of developing PD (adjusted OR = 0.204, 95% CI = 0.047–0.886, *p* = 0.0399) [52]. Their findings suggested that dental scaling in individuals without periodontitis had a greater protective effect than in those with periodontitis, and this tendency was more predominant in the group aged 40 to 69 year compared to 70 years. A study from South Korea evaluated the effects of one year of periodontitis treatment on the incidence of PD. The risk was found to be higher for patients who required further visits [40], i.e., HR of PD incidence 1.142 (95% CI 1.094–1.193). Results from both large-scale studies combined with emerging data from association studies suggest the plausibility of periodontitis as a modifiable risk factor for PD. In addition to conventional treatment, adjuvant therapy of periodontitis with laser and photoactivation, although not part of the current clinical recommendations [10] may also be considered due to its beneficial effect on oxidative stress [53]. Oxidative stress plays a role in neuroinflammation [3]. In a recent intervention study in PD, application of photodynamic therapy resulted in significant improvement in the clinical parameters of periodontitis and immunological biomarkers IL-6 and TNF-α in PD patients with severe periodontitis [54]. All these therapeutic modalities need to be explored further.

### 3.3. Common Risk Factors

Periodontitis and neurodegenerative diseases share many risk factors. These are presented in Figure 2 and include age, gender, race, smoking and alcohol intake, nutrition, physical activity, socioeconomic status, stress, medical comorbidities and genetics [33]. In view of these associations, the relationship between periodontitis and neurodegenerative disease can be challenging to examine, as they may act as confounding variables. A recent systematic review demonstrated that even in large cross-sectional studies with sufficient sample sizes, none of the conducted studies corrected for all anticipated confounding variables [34].

## 4. What Is the Plausible Mechanistic Link for the Association Neurodegenerative Diseases and Periodontitis?

An increasing amount of research recognizes the interplay between periodontal inflammation and neuroinflammation as plausible mechanistic link for the association periodontitis and neurodegenerative diseases. Neuroinflammation is defined as the process of triggering the brain’s innate immune system after an immunological challenge caused by an injury, infection, neurodegenerative disease, aging or exposure to bacteria or their metabolites [55]. Subsequent to the activation of the immune system, microglia sense the possible homeostatic imbalance and a multitude of biochemical and cellular activities are triggered, including activation of astrocytes [56]. This protective upregulation of inflammatory signals by the microglia to protect the central nervous system (CNS) can, however, also yield harmful effects as an inflammatory response becomes chronic and damaging. If the systemic inflammatory stimulus is not resolved promptly, it can result in a condition where constantly activated microglia become excessively sensitive to a new immune trigger and respond disproportionately. Individuals diagnosed with periodontitis may experience neuroinflammation as a result of prolonged systemic inflammation [3].

### 4.1. Central Nervous System (CNS)

Astrocytes, the predominant neural type of glia in the CNS, and microglial cells, deriving from the hematopoietic lineage with monocyte/macrophage precursors are distributed uniformly throughout the parenchyma of the CNS [57]. They work closely together in both normal and pathological conditions. Astrocytes are not immune cells. However, with their intricate branching structure, extending long and thin processes they envelop neuronal processes and synapses. This morphology enables them to perform various important functions, such as regulating neuronal activity, providing metabolic support to neurons, maintaining the balance of extracellular fluids, and isolating excitable cells electrically [58]. Activated microglial cells undergo morphological changes and display increased proliferation, acquiring of migration capacity through amoeboid motility, increased phagocytic activity. Furthermore, they secrete pro-inflammatory cytokines (IL-1β, IL-6, TNF-α) and cytotoxic compounds such as reactive oxidative species (ROS) [57]. The increased level of ROS can cause neurotoxicity that affects several proteins and homeostasis in the cells [3]. Simultaneously, astrocytes also undergo proliferation and secrete matrix components, leading to the formation of a “glial scar” surrounding the affected area. Additionally, these astrocytes modify their secretome, now producing pro-inflammatory and chemotactic mediators [59]. Also contributing to the processes involved in neuroinflammation are the neurons, through constant communication with microglia, endothelial cells and pericytes of CNS blood vessels [60].

### 4.2. Chronic Neuroinflammation and Pathogenesis of Neurodegenerative Disorders

To date, the exact cause of the most prevalent neurodegenerative disorders, such as AD and PD, is still unknown. However, chronic neuroinflammation is increasingly recognized as a potentially significant factor in these disorders. Furthermore, evidence indicates that systemic inflammation may potentially trigger the development of neuroinflammation.

Research suggests that periodontitis may play a role in the development or progression of AD through various pathways, including the involvement of specific periodontal pathogens, such as *P. gingivalis*. In individuals with AD, it is believed that an excess of activated microglia fails to effectively remove amyloid-beta plaques through phagocytosis, thereby contributing to their accumulation [61,62].

Neuroinflammation is also recognized as a critical component in PD. It is thought that an inflammatory response in the gut may be responsible for initiating the disease. It has been hypothesized that bacteria form the gut could access the brain via the vagus nerve, subsequently leading to inflammation in the brain, particularly in the substantia nigra region which disrupts the production of dopamine—a hallmark feature of PD [61,62].

Neuroinflammation is also believed to play a crucial role in the onset and progression of MS. Research indicates that the presence of inflammatory cytokines disrupts the blood–brain barrier, allowing B cells and plasma cells to enter the central nervous system. These immune cells then damage the myelin sheath, leading to demyelination, a primary symptom of the disease [61,62].

### 4.3. Pathogenesis of Periodontitis and Its Role in Neuroinflammation

In periodontitis, oral microbial dysbiosis triggers exaggerated chronic inflammation in susceptible individuals. As mentioned previously, in the current model of pathogenesis of periodontitis microbial dysbiosis and local inflammation co-develop in a reciprocally reinforced manner [11,12]. Microbial communities of key stone pathogens and pathobionts with synergetic virulence withstand the host response and propagate by inducing tissue-destructive inflammation medicated by the host immune system. In this way, a self-sustaining vicious circle of escalating dysbiosis and uncontrolled inflammation arises, which if not broken can lead to tooth loss and systemic complications.

**Figure 3:**
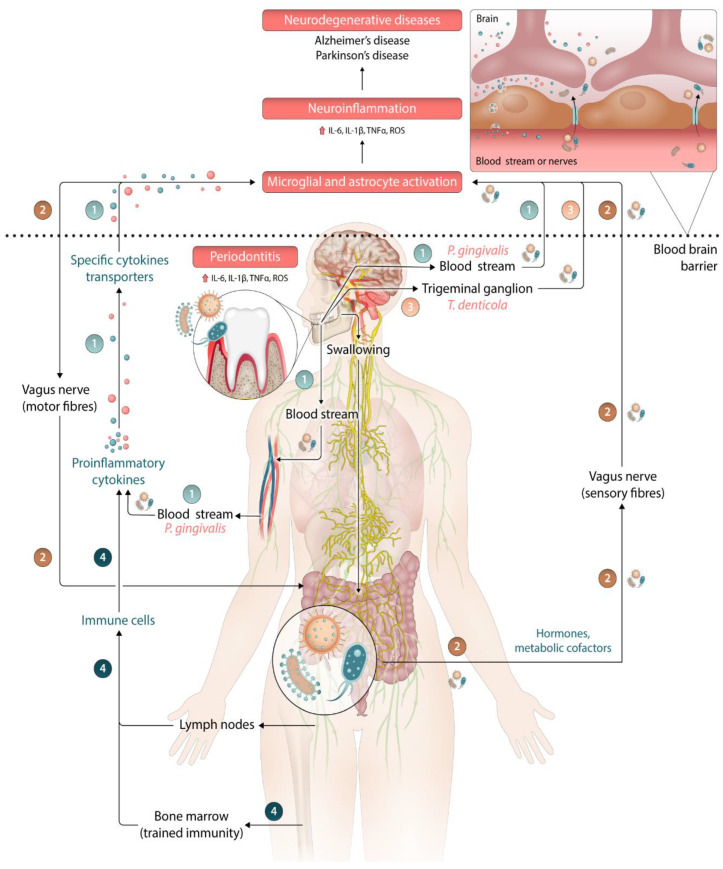
Hypothetic schematic representation of how periodontitis could stimulate neuroinflammation leading to neurodegenerative diseases based on current literature [24,25,26,27,28,29,60]. The following major pathways and mechanisms are suspected: (1) proinflammatory cytokines reach the brain parenchyma via the bloodstream; (2) oral–gut–brain axis and vagus nerve pathway; (3) bacteria travel to the brain via the trigeminal nerve and (4) trained immunity. The arrow ⇑ indicates increased levels of cytokines or reactive oxydative species (ROS).

Currently, potential direct and indirect mechanisms through which periodontitis may contribute to the development and progression of neurodegeneration diseases have been described in the literature. In summary, humoral, neuronal and cellular pathways have been hypothesized with clear role of the periodontal pathogens that can exert their effects via different routes as presented in Figure 3:

(1) Proinflammatory cytokines locally released in periodontitis or induced by the low-grade systemic inflammation can enter the brain via the bloodstream.
(2) Periodontal pathogens can reach the guts via the bloodstream or by swallowing. These have the potential to disrupt the gut microbiota. The inflamed gut epithelial cells release proinflammatory mediators that travel through the bloodstream and enter the brain, i.e., “oral–gut–brain axis”. Another way to enter the brain is via the vagus nerve.
(3) Periodontal pathogens can reach the brain through the trigeminal nerve [60].
(4) Periodontal pathogens might trigger trained myelopoiesis (trained immunity) that might induce with hyperinflammatory response that could affect the brain.

An increasing number of preclinical studies try to further elucidate these plausible pathogenic mechanisms. *P. gingivalis*, being the “key stone pathogen” in the etiopathogenesis of periodontitis, is currently the most extensively studied bacteria in neuroinflammation. Animal studies routinely demonstrate its migration from the oral cavity to the CNS, and post-mortem human studies in AD confirm regularly its presence intracerebrally [20,28]. *P. gingivalis* receives this special attention due to its unique ability to escape from the immune system, its invasive properties, proteolytic nature and aggressive virulence factors, such as LPS and gingipains. There is some evidence that *P. gingivalis* and its gingipains can directly invade astrocytes, microglia and neurons and mediate their neuroinflammatory activity [63]. In addition, gingipains appears to be capable to initiate physical injury to the cerebral microvasculature with degeneration of endothelial tight junctions and increased permeability of the blood–brain barrier (BBB). Based on the findings of animal studies the concept of 10-year lag phase for periodontitis to become a risk factor for the development of AD has been suggested [64,65,66].

In addition to *P. gingivalis*, other periodontal pathogens, such as *T. denticola*, *Aggregatibacter actinomycetemcomitans (A. actinomycetemcomitans)*, and *F. nucleatum*, have received attention for their potential role in neuroinflammation, especially in AD [67]. Some interesting findings from animals studies are that *Treponema* species appear to have the ability to invade CNS and produce amyloid, serotype b of *A. actinomycetemcomitans* triggers secretion of proinflammatory cytokines by microglia and *F. nucleatum*-induced periodontitis can lead to the worsening of Alzheimer symptoms in mice [68]. In all cases with translocated periodontal pathogen to distant sites, including post-mortem studies in AD, there remains the critical question: are these bacteria “passengers” or “promoters” as detection of oral bacteria in extraoral cites does not necessarily mean pathogenicity.

Finally, the theoretical concept of “oral–gut–brain axis” (Figure 3) needs some further explanation. It is a complex system that relies on the gut microbiome to regulate various neurochemical pathways. Disruption of this system can disrupt homeostasis. The blood–brain barrier (BBB) and the blood cerebrospinal fluid (CSF) barriers play a vital role in controlling neuroinflammation. The gut can impact the BBB through the secretion of hormones, metabolic cofactors, and the production of small molecules. Additionally, inflammatory mechanisms such as cytokine or oxidative stress can affect BBB permeability. Oral microbiome could affect both the gum microbiome and the BBB directly due to anatomical connections, and indirectly by immunological response. This complex interplay suggests the existence of an “oral–gut–brain axis”, which is of special interest for the pathogenesis of PD.

## 5. Summary and Final Conclusions

Currently, AD is the most frequently reported neurodegenerative disease to be associated with periodontitis, and this association is overall positive. For PD, this association appears to be weak. Nevertheless, there is emerging evidence for an association between poor oral health and PD.

Patients with periodontitis appear to have an increased risk for AD and MCI. AD, PD, and MCI patients may have poor oral health and be at risk for developing periodontitis. Cognitive decline and physical disability play also role in it. Reverse causality cannot be excluded. Age, gender, race, smoking/alcohol intake, nutrition, physical activity, socioeconomic status, stress, medical comorbidities and genetics are identified as common risk factors for periodontitis and neurodegenerative diseases.

Chronic neuroinflammation triggered by systemic inflammation is the plausible mechanistic link. It is hypothesized that periodontitis can induce chronic systemic inflammation, which can affect the brain via humoral, neural and cellular pathways. The concept of “oral–gut–brain axis” has emerged. For AD, a bidirectional relationship with periodontitis is suspected. For PD, taking into consideration the huge knowledge gap, bidirectional relationship could be speculated.

Finally, we should mention the strengths and limitations of our literature review. We applied a systematic search strategy in three databases that allowed us to present a comprehensive overview of the best available evidence on association between periodontitis and neurodegenerative diseases. In addition, we included and discussed many other relevant articles to elucidate the plausibility of bidirectional relationships and provide insight into pathogenetic mechanisms. This has increased the scope of this review, and is the strength of our study. However, we did not provide qualitative or quantitative analyses of the original results on association, which could be considered as a limitation. An umbrella review instead of a narrative review could have strengthened our conclusions. Still, our work has additional value to the umbrella review of Wang et al. [32]. We used a broader search strategy and included systematic reviews not only restricted to the microbiological factors of periodontitis as a link to neurodegenerative diseases. The diagnosis of periodontitis is primarily clinical and not always and necessarily supported by microbiological findings. A research aim restricted to microbiological factors might fail to identify important clinical studies on this topic. We also addressed the issue of common risk factors between periodontitis and neurodegenerative diseases. This is critical for such complex diseases, especially when discussing associations between them. In this way, we have provided more complete overview on the current research and have drawn attention to study factors that can impact these associations. Nevertheless, we did not discuss other issues, such as different diagnostic criteria used for periodontitis and neurodegenerative diseases, which can also influence study outcomes. Also, studies on decline in dental care in patients with neurodegenerative disorders and their potential effect on the periodontium were not highlighted enough. Instead, we focused on summarizing the evidence for an association, elucidating pathogenesis and discussing periodontitis as a modifiable risk factor for neurodegenerative diseases. Despite our systematic and comprehensive approach, the potential of periodontitis and its therapy to prevent or slow down neurodegenerative diseases might not be fully explained, and has to be explored further and in more detail.

## 6. Directions for Future Research

Our findings offer a clear recommendation to both neurologists and dentists working in this field: It is high time to join forces to fill the knowledge gap and discover a possible way to prevent neurodegenerative diseases, or to modify their relentless progressive course. It is evident that poor oral health is a common problem in neurodegenerative diseases, and it is time to better understand its significance. It is important to screen for periodontitis in patients with increased risk or with prodromal symptoms of AD and PD in view of the hypothesized bidirectional relationship and treat as early as possible in view of plausible protective effect. At the same time, potential reverse causality should be considered. Periodontitis may be not the game changer, but it could change the game.

Ironically, a similar call for interdisciplinary cooperation as future direction was given nearly 100 years ago by Roland P. Mackay, a giant in American neurology. Back in 1937, he said, “*As far as the fields of dentistry and neurology are concerned, they should be especially close […] I am convinced that every neurologist should know more dentistry, and that the average dentist would profit in many ways if he had more information about neurology*” [69]. Obviously, despite progress in research, history tends to repeat itself. Evidently, increasing scientific knowledge does not necessarily mean increasing human wisdom (i.e., intelligence). Perhaps artificial intelligence could help to take action. So, let us finish optimistically, with “hopamine”, but still keep in mind that artificial intelligence may not beat human stupidity.

## Figures and Tables

**Table 1:** High-level evidence studies published during the last 5 years selected for discussion.

Reference	Design	Neurodegenerative Disease	Research Aim	Number of Studies	Type of Included Studies	Sample Size	Main Findings
**Leira et al. 2017** [25]	SR + MA	**Alzheimer’s disease**	To evaluate the association between periodontal disease and AD	**5**	2 cross-sectional, 2 case–control 1 cohort study	982	Periodontal disease is significantly associated with the presence of AD: OR = 1.69 (95% CI 1.21–2.35); for severe periodontal disease and AD: OR = 2.98 (95% CI 1.58–5.62)
**Alvarenga et al. 2021** [26]	SR	**Alzheimer’s disease Parkinson’s disease, Multiple Sclerosis**	To evaluate the association between neurodegenerative disorders and periodontitis	**12**	8 case–control 3 cross-sectional 1 cohort	3460	All included studies reported an association with periodontitis. The level of evidence was very low due to heterogeneity of the studies.
**Hu et al. 2021** [27]	SR + MA	**Alzheimer’s disease** **Mild cognitive impairment**	To evaluate the association between periodontal disease and the risk of AD or MCI	**13**	MA on 8 studies on AD: cross-sectional case–control cohort MA on 8 studies on MCI: cross-sectional cohort	291, 114	The risk of AD and MCI in patients with periodontitis was significantly higher compared to the non-periodontal disease population. for AD: OR = 1.78 (95% CI 1.15–2.76) for MCI: OR = 1.60 (95% CI 1.24–2.06) for severe periodontal disease and AD: OR = 4.89 (95% CI 1.60–14.97) severe periodontal disease and MCI: OR = 2.32 (95% CI 1.24–4.36)
						4805	
**Borsa et al. 2021** [19]	SR	**Alzheimer’s disease**	To evaluate the association between Alzheimer’s disease and periodontal disease in patients aged 65 and over	**5**	3 case–control 1 cross-sectional 1 cohort	59 to 3251	An increase in *F. nucleatum* in AD patients (adjusted *p* = 0.02) Incidence of AD linked to *C. rectus*, *P. gingivalis* (adjusted HR = 1.22 (1.04–1.43), *p* = 0.012) and *A. naeslundii* (crude HR = 2.0 (1.1–3.8) Presence of periodontitis at baseline was associated with a six-fold increase in rate of cognitive decline over a 6-month follow-up (ADAS-Cog mean change = 2.9 ± 6.6)
**Mao et al. 2022** [28]	SR	**Alzheimer’s disease**	To evaluate the association between the oral microbiome and AD development by using the next-generation sequencing technique	**26**	7 cross-sectional 7 cohort 7 case–control 5 post-mortem Studies were divided in: − databased − periodontal − serology − microbiology − post-mortem	219 to 27,963 85 to 951 34 to 158 20 to 195 20 to 58	Presence of periodontitis is associated with AD; Gram-negative species may be possible candidates. There is a possible role of Gram-negative periodontal pathogens in the pathogenesis of AD
**Chen et al. 2023** [29]	SR + MA	**Parkinson’s disease**	To evaluate bilateral relationship between PD and periodontitis	**34**	6 cohort 16 case–control 11 cross-sectional 1 Mendelian randomization (MR) study MA on 24 studies	7720, 793	No significant association with PD risk (HR = 1.13, 95% CI 0.88–1.45, n = 3; OR = 1.94, 95% CI 0.55–6.90, n = 7) Patients with PD have poor oral health PD patients exhibited higher levels of periodontal pocket depth (SMD = 1.10, 95% CI 0.53–1.67), clinical attachment level (SMD = 1.40, 95% CI 0.55–2.26), plaque index (SMD = 0.81, 95% CI 0.22–1.39), and Oral Health Impact Profile-14 score (SMD = 0.91, 95% CI 0.33–1.49) compared to healthy controls

Abbreviations used: systematic review (SR); meta-analysis (MA); Alzheimer’s disease (AD), Parkinson’s disease (PD), and mild cognitive impairment (MCI).
